# A narrative review on perioperative systemic therapy in non-small cell lung cancer

**DOI:** 10.37349/etat.2024.00256

**Published:** 2024-07-26

**Authors:** Robert Hsu, Zhaohui Liao Arter, Darin Poei, David J. Benjamin

**Affiliations:** Istituto Nazionale Tumori-IRCCS-Fondazione G. Pascale, Italy; ^1^Division of Medical Oncology, University of Southern California Norris Comprehensive Cancer Center, University of Southern California Keck School of Medicine, Los Angeles, CA 90033, USA; ^2^Department of Medicine, Division of Hematology-Oncology, University of California Irvine School of Medicine, Orange, CA 92697, USA; ^3^Department of Medicine, University of Southern California Keck School of Medicine, Los Angeles, CA 90033, USA; ^4^Hoag Family Cancer Institute, Newport Beach, CA 92663, USA

**Keywords:** Non-small cell lung cancer, perioperative, neoadjuvant, adjuvant, immunotherapy, targeted therapy

## Abstract

Non-small cell lung cancer (NSCLC) that is operable still carries a high risk of recurrence, approaching 50% of all operable cases despite adding adjuvant chemotherapy. However, the utilization of immunotherapy and targeted therapy moving beyond the metastatic NSCLC setting and into early-stage perioperative management has generated tremendous enthusiasm and has been practice-changing. Adjuvant atezolizumab in NSCLC first demonstrated a clinical benefit with an immune checkpoint inhibitor. Then, with studies studying a significant benefit in major pathologic response in surgical patients treated preoperatively with immunotherapy compared to only chemotherapy, neoadjuvant nivolumab and chemotherapy were evaluated and showed significant event-free survival benefit leading to subsequent studies evaluating perioperative immunotherapy and chemotherapy. Meanwhile, with regards to targeted therapies, adjuvant osimertinib in *EGFR*-mutated NSCLC and adjuvant alectinib in *ALK*-rearranged NSCLC have both received regulatory approvals following demonstrated clinical benefit in clinical trials. With rapidly evolving changes in the field, new combinations such as multiple immunotherapy agents and antibody-drug conjugates in development, perioperative NSCLC management has quickly become complicated with different pathways to perioperative treatment. Furthermore, circulating tumor DNA and studies looking at better tools to prognosticate immunotherapy response will help with decision-making regarding which patients should receive immunotherapy and if so, either only pre-operatively or both pre- and post-operatively. In this review, we look at the evolution of systemic therapy in the perioperative setting from adjuvant chemotherapy to adjuvant immunotherapy to perioperative immunotherapy and look at perioperative targeted therapy while looking ahead to future considerations.

## Introduction

For a long time, perioperative non-small cell lung cancer (NSCLC) management from a systemic therapy standpoint was relatively simple. There was a benefit in overall survival (OS) for stage II–III NSCLC cases to receive adjuvant cisplatin-based chemotherapy per the meta-analysis performed by the Lung Adjuvant Cisplatin Evaluation (LACE) study [1]. However, systemic therapy in NSCLC has advanced significantly over the past 10–15 years with the use of immune checkpoint inhibitors (ICIs) and the development of targeted therapies for specific mutations in NSCLC. Consequently, there have been notable developments since then incorporating adjuvant systemic therapies, neoadjuvant systemic therapies, and combination therapies with treatment given both in the neoadjuvant and adjuvant settings. With regards to perioperative use of ICIs, the key first studies involved adjuvant atezolizumab based on findings in the IMPower010 for programmed death-ligand 1 (PD-L1) tumor proportion score (TPS) of ≥ 1 along with KEYNOTE 091 which evaluated adjuvant pembrolizumab [2, 3]. Eventually, preoperative immunotherapy was looked at in a pivotal pilot study of 20 patients receiving nivolumab which set the stage for the use of neoadjuvant chemotherapy with nivolumab as per the CheckMate-816 study [4, 5]. Ultimately, this led to perioperative immunotherapy trials such as pembrolizumab in early-stage NSCLC per the KEYNOTE-671 study and nivolumab per the Checkmate-77T study [6, 7]. Meanwhile, perioperative studies for oncogenic-driven NSCLC have been done, including epidermal growth factor receptor (*EGFR*) mutations and anaplastic lymphoma kinase (*ALK*) rearrangement. Notably, the ADAURA study evaluated 3 years of adjuvant osimertinib in resected stage IB–IIIA *EGFR* Exon 19 deletion or L858R NSCLC and similarly, the ALINA trial looked at the use of adjuvant alectinib for two years in *ALK* rearranged NSCLC, both of which led to regulatory approvals and are part of standard of care management [8–11]. In our review, we discuss these developments and consider new combinations and tools that may augment the current response rates and better inform clinicians about deciding when to use immunotherapy and targeted therapy in the perioperative setting.

## Methods

For this narrative review, we included studies that based on the authors’ knowledge and expertise in the thoracic medical oncology field were determined to be practice-changing with a focus on studies that had been approved by regulatory agencies such as the United States Food and Drug Administration (FDA). From these studies, we did a PubMed search to identify these articles in the English language. For studies on drugs that were still in development and had not been published as a manuscript, we used the Google search engine to identify recent findings that had been presented at oncology conferences.

## Adjuvant chemotherapy

NSCLC has a high risk of recurrence in about 30–55% of NSCLC patients who receive curative resection [12]. An initial large meta-analysis was performed in 1995 consisting of 52 randomized clinical trials involving 9,387 patients and suggested a possible 5-year OS benefit, but the difference in OS was not statistically significant. Therefore, additional randomized clinical trials investigated adjuvant cisplatin chemotherapy regimens were performed [13]. These studies included the International Adjuvant Lung Cancer Trial which showed an improved 5-year survival rate [44.5% vs. 40.4%, hazard ratio (HR) = 0.86, 95% confidence interval (CI): 0.76–0.98] along with Adjuvant Navelbine Trialist Association Trial [median OS: 65.7 months (95% CI: 47.9–88.5) vs. 43.7 months (95% CI: 35.7–52.3)]. However, the Big Lung Trial showed no statistical OS benefit in stage I–III NSCLC surgical patients receiving chemotherapy with a study led by the Adjuvant Lung Project Italy/European Organization for Research Treatment of Cancer-Lung Cancer Cooperative Group, which showed no statistical benefit in 1,209 stage I–III surgically NSCLC patients who received mitomycin C, vindesine, and cisplatin for 3 cycles [OS HR = 0.96 (95% CI: 0.81–1.13), *P* = 0.589] [14–17].

Subsequently, the LACE was a pooled analysis that consisted of randomized trials composed of more than 300 patients comparing cisplatin-based chemotherapy vs. no chemotherapy or cisplatin-based chemotherapy plus postoperative radiotherapy vs. postoperative radiotherapy alone in patients with completely resected NSCLC [1]. This analysis ultimately consisted of five trials with 4,584 patients, which consisted of the JBR10 trial involving patients with pathologic tumor stage 2 pathologic nodal stage 0 (pT2-pN0) or pT1-2pN1 and then received 4 adjuvant cycles of cisplatin and vinorelbine along with the Adjuvant Lung Project Italy/European Organization for Research Treatment of Cancer, Adjuvant Navelbine International Trialist Association, International Adjuvant Trial, and the Big Lung Trial (excluding 74 patients who received neoadjuvant chemotherapy) [1, 14–18].

There was a statistically significant OS benefit (HR = 0.89, 95% CI: 0.82–0.96, *P* = 0.005) for adjuvant chemotherapy vs. no chemotherapy. There was a 3.9% absolute benefit at 3 years survival, a 5.4% absolute OS benefit at 5 years, and a 5.8% disease-free survival (DFS) benefit at 5 years. By stage stratification, there was clear benefit for adjuvant chemotherapy in stage II (OS HR = 0.83, 95% CI: 0.73–0.95) and stage III resected NSCLC (OS HR 0.83, 95% CI: 0.72–0.94) but not in stage IA (OS HR = 1.40, 95% CI: 0.95–2.06) nor stage IB (OS HR = 0.93, 95% CI: 0.78–1.10) [1].

With regard to high-risk features such as visceral pleural invasion (VPI), there has been little definitive evidence of the benefit of adjuvant chemotherapy. VPI has been shown to be a poor prognostic indicator; in stage IB patients with T2 VPI, the 5-year and 10-year OS was 44% and 28 % respectively compared to 63% and 60% in the pleural non-invasion group [19]. However, there have been mixed results as a Korean retrospective study in which patients in the adjuvant chemotherapy group showed a significantly reduced recurrence rate and risk of mortality than those in the non-adjuvant chemotherapy group but Surveillance, Epidemiology, and End Results (SEER) data from 2004–2015 of 1,993 NSCLC patients with peripheral tumors with VPI and tumors 3 cm or less did not show survival benefit to adjuvant chemotherapy [20, 21]. However, more recent analysis using cancer registry database data has highlighted two specific high-risk factors—tumor size and differentiation of the tumor. Cheng et al. [22] performed a retrospective cohort study from 26,380 SEER database patients with pathological N0 NSCLC after surgery and showed that adjuvant chemotherapy showed a benefit in 2-year OS in T2bN0 patients but not in T2aN0 NSCLC patients; adjuvant chemotherapy led to an improvement in 24 months survival in poorly differentiated NSCLC (86.36% vs. 81.70%, *P* = 0.029) and in tumor sizes larger than 4 cm. Meanwhile, a National Cancer Database study of 50,814 treatment-naive patients with a completely resected node-negative NSCLC diagnosis from 2010–2015 showed no benefit in tumors 3 cm or smaller, a significant benefit in patients with tumors 4–5 cm receiving sublobar surgery, and in patients with tumors greater than 5 cm with a high-risk pathologic feature such as VPI, lymphovascular invasion, or high-grade histologic finding [23].

Currently, the National Comprehensive Cancer Network (NCCN) guidelines recommend that adjuvant chemotherapy be given to all patients with stage IIB along with stage IIIA and stage IIIB NSCLC with negative margins [24]. For stage IIA (T2bN0) and negative margins, the NCCN guidelines recommend adjuvant chemotherapy for high-risk features, which include poorly differentiated tumors, vascular invasion, wedge resection, VPI, and unknown lymph node status [24].

## Adjuvant immunotherapy

Immunotherapy, specifically ICIs, plays an integral role in NSCLC therapy in patients without actionable mutations such as *EGFR*, *ALK*, rearranged during transfection (*RET*), and ROS proto-oncogene 1 (*ROS1*) mutations; in these specific mutations, previous studies such as IMMUNOTARGET registry study have shown less ICI efficacy [25]. ICI therapy in clinical application has primarily consisted of the PD-L1 and cytotoxic T lymphocyte antigen 4 (CTLA-4) pathway [26]. ICI therapy was first shown to have benefits in the stage IV NSCLC in the second-line with studies comparing nivolumab and pembrolizumab, programmed death 1 (PD-1) inhibitors along with atezolizumab, a PD-L1 inhibitor, with docetaxel and showing significant overall response rate (ORR) and OS benefit [27–30]. This ultimately then led to multiple front-line strategies in stage IV NSCLC including immunotherapy monotherapy in PD-L1 tumor proportion score ≥ 50%, chemotherapy with immunotherapy, dual ICI therapy with PD-1/PD-L1 and CTLA-4 inhibitor therapy [31–36]. The success of ICI use led towards considering ICI in locally advanced NSCLC, as the PACIFIC trial evaluated unresectable stage III NSCLC comparing durvalumab vs. placebo in patients who received chemotherapy and radiation before durvalumab or placebo and showed a 5-year OS of 43% in patients receiving durvalumab vs. 33% in patients receiving placebo [37, 38].

In resectable NSCLC, it was believed that cancer surgery-induced immune dysfunction may provide an immune microenvironment conducive to ICI therapy, as surgical trauma may increase inflammatory cytokines such as interleukin-10 (IL-10), tumor necrosis factor (TNF)-alpha, and IL-6/8 and also increase growth and clotting factors and stress hormones leading towards the expansion of regulatory T cells (Tregs), myeloid-derived suppressor cells (MDSCs), M2 macrophages, and PD-1/CTLA-4 expression and so ICI effect can block the binding of PD-1/PD-L1 and upregulate the growth and proliferation of T cells leading to an antitumor effect [39, 40].

Thus, IMPower010 was a pivotal trial in which patients with resected stage IB–IIIA NSCLC received 1 year of adjuvant atezolizumab after chemotherapy [41]. At median 45.3 months follow up, atezolizumab significantly improved DFS vs. best supportive care after resection and adjuvant chemotherapy in PD-L1 tumor cell (TC) ≥ 1% and OS benefit with atezolizumab vs. best supportive care being strongest in the PD-L1 ≥ 50% in stage II–IIIA HR = 0.43 (95% CI: 0.24–0.78). No OS improvement in favor of atezolizumab was seen vs. best supportive care in the intention to treat population or the stage II–IIIA populations though OS data has not matured yet. There were no new safety signals seen; immune-mediated adverse events occurred in 52.1% with grade 3 or 4 immune-mediated adverse events seen in 7.9% of patients. Grade 5 immune-mediated pneumonitis and myocarditis were seen in 2 (0.4%) patients [42]. Another study, KEYNOTE 091, also looked at adjuvant pembrolizumab for 1 year following optional adjuvant chemotherapy and interim analysis showed a median DFS benefit in the pembrolizumab arm (53.6 months, 95% CI: 39.2–not reached) vs. placebo arm (42.0 months 95% CI: 31.3–not reached). Similarly, pembrolizumab was well tolerated with serious adverse events in 24% of patients and treatment-related adverse events leading to death in 4 (1%) patients treated with pembrolizumab (one due to cardiogenic shock and myocarditis, one due to septic shock, and myocarditis, one due to pneumonia, and one due to sudden death) [3]. Of note, both trials did not exclude patients who had *EGFR* or *ALK* mutations.

Other notable pending studies include the ANVIL study in which patients receive adjuvant nivolumab for one year after optional chemotherapy or radiotherapy (NCT02595944), the ALCHEMIST Chemo-IO (NCT04267848) study in which patients without *EGFR* mutations or *ALK* rearrangements will either receive chemotherapy-PD-1 inhibition with pembrolizumab during and after, sequential chemotherapy followed by pembrolizumab or chemotherapy alone, BR.31 study (NCT02273375) looking at adjuvant durvalumab in completed resected NSCLC, MERMAID-1 (NCT04385368) in which patients will receive adjuvant durvalumab and chemotherapy or placebo and chemotherapy and the primary outcome measure is DFS in the minimal residual disease (MRD)-positive analysis set (defined as patients with positive circulating tumor DNA (ctDNA) 3–4 weeks post-surgery), and the MERMAID-2 (NCT04642469) in which patients after resection will be monitored for MRD via detection of ctDNA and those who become MRD-positive during the surveillance period with no visible disease recurrence will be randomized 1:1 to receive adjuvant durvalumab or placebo every 4 weeks with the primary endpoint being DFS in patients with PD-L1 TPS ≥ 1% (Table 1).

**Table 1:** Ongoing phase III clinical trials of perioperative immunotherapy with PD-1/PD-L1 inhibitors in operable NSCLC

**Trial**	**Stage**	**Treatment**	**Control**	**Primary endpoint**	**Primary Outcome**
Neoadjuvant: CheckMate 816 [5]	IB–IIIA	Nivolumab + chemotherapy × 3 cycles	Chemotherapy	EFS	EFS: 31.6 months vs. 20.8 months
Adjuvant: IMpower010 [2]	IB (> 4 cm)–IIIA	Chemotherapy → atezolizumab 16 cycles	Chemotherapy → observation	DFS	DFS: HR = 0.81 (0.67–0.99)
Keynote-091 [3]	IB (> 4 cm)–IIIA	Chemotherapy (optional) → pembrolizumab 18 cycles	Chemotherapy (optional) → placebo	DFS	mDFS: 53.6 months vs. 42 months [HR = 0.76, 95% CI: 0.63–0.91]
BR.31 (NCT02273375)	IB (> 4 cm)–IIIA	Chemotherapy(optional) → durvalumab 12 months	Chemotherapy (optional) → placebo	DFS	N/A
ANVIL (NCT02595944)	IB (> 4 cm)–IIIA	Chemotherapy(optional) → nivolumab 16 cycles	Chemotherapy (optional) → observation	DFS, OS	N/A
MERMAID-1 (NCT04385368)	II–III	Durvalumab + SoC chemotherapy	Placebo + SoC chemotherapy	DFS	N/A
MERMAID-2 (NCT04642469)	II–III	Durvalumab 1 year	Placebo	DFS	N/A
ALCHEMIST (NCT04267848)	IB (> 4 cm)–IIIA	Chemotherapy → pembrolizumab 16 cycles; or chemotherapy + pembrolizumab 4 cycles → pembrolizumab 12 cycles	Chemotherapy → observation	DFS, OS	N/A
Perioperative: KEYNOTE-671 [43]	II–IIIA	Neoadjuvant Pembrolizumab + chemotherapy 4 cycles; adjuvant Pembrolizumab	Neoadjuvant chemotherapy; adjuvant placebo	EFS, OS	EFS at 24 months: 62.4% vs. 40.6% (HR = 0.58, 95% CI: 0.46–0.72); OS at 24 months: 80.9% vs. 77.6% (*P* = 0.02)
CheckMate-77T [7]	II–IIIB	Neoadjuvant nivolumab + chemotherapy 4 cycles; adjuvant nivolumab	Neoadjuvant chemotherapy; adjuvant placebo	EFS	EFS at 18 months: 70.2% vs. 50.0% (HR = 0.58, 97.36% CI: 0.42–0.91)
IMpower030 [44]	II–IIIB	Neoadjuvant Atezolizumab + chemotherapy 4 cycles; adjuvant atezolizumab 16 cycles	Neoadjuvant chemotherapy; adjuvant monitoring	EFS	N/A
AEGEAN [45]	IIA–IIIB	Neoadjuvant durvalumab + chemotherapy 4 cycles; adjuvant durvalumab 12 cycles	Neoadjuvant chemotherapy; adjuvant placebo	EFS, PCR	EFS at 12 months: 73.4% vs. 64.5% (HR = 0.68, 95% CI: 0.53–0.88); PCR: 17.2% vs. 4.3% (95% CI: 8.7 to 17.6)
RATIONALE-315 [46]	II–IIIA	Neoadjuvant tislelizumab + chemotherapy 3–4 cycles; adjuvant tislelizumab up to 8 cycles	Neoadjuvant chemotherapy; adjuvant placebo	EFS	Median EFS was not reached at 22 months for either arm; however, a statistically significant difference in EFS (HR = 0.56, 95% CI: 0.40–0.79)
JS001-029	IIIA	Neoadjuvant toripalimab + chemotherapy 4 cycles; adjuvant toripalimab 13 cycles	Neoadjuvant chemotherapy; adjuvant placebo	MPR, EFS	N/A
NCT05157776	IIIA	Neoadjuvant sintilimab + chemo 4 cycles	Neoadjuvant sintilimab + chemotherapy 2 cycles; adjuvant: optional Sintilimab + chemotherapy 2 cycles	PCR	N/A

DFS: disease-free survival; EFS: event-free survival; MPR: major pathological response; OS: overall survival; PCR: pathologically complete response; N/A: not applicable; HR: hazard ratio; CI: confidence interval; OS: overall survival; PD-1: programmed death 1; PD-L1: programmed death-ligand 1; NSCLC: non-small cell lung cancer

## Neoadjuvant immunotherapy

Neoadjuvant immunotherapy involves the administration of ICIs specifically PD-1/PD-L1 blockade before surgical resection in patients with NSCLC. The rationale behind this approach is multifaceted: it aims to reduce tumor size, potentially enabling less extensive procedures, and targeting undetectable metastatic disease early on. This approach not only primes the immune system by exposing it to tumor antigens, enhancing the body’s natural defense against cancer but also allows for the evaluation of treatment response through pathological assessment of the resected tumor.

At this stage, anti-PD-1 therapy can stimulate the growth of T-cell clones in the peripheral blood that are specific to mutation-associated neoantigens [47]. A pilot study of neoadjuvant PD-1 blockade in resectable NSCLC led by Forde et al. [4] showed a major pathological response defined as at least 90% regression in 9 of 20 (45%) resected tumors. This was a significant improvement from previous neoadjuvant chemotherapy-only studies in which the median pathological complete response (PCR) from 15 trials was 4% (range 0–16%) providing the rationale for the CheckMate 816 trial [48].

The CheckMate 816 trial was a phase III study that investigated the efficacy and safety of combining nivolumab, a PD-1 inhibitor, with platinum-doublet chemotherapy in patients with resectable NSCLC. In this study, patients either received nivolumab plus platinum-based chemotherapy for 3 cycles or platinum-based chemotherapy alone in a 1:1 randomization followed by resection. The primary endpoints were PCR and event-free survival (EFS), with secondary endpoints focusing on OS and safety profiles [5]. CheckMate 816 demonstrated a notable improvement in PCR rates among patients receiving the nivolumab-chemotherapy combination compared to chemotherapy alone [24.0% (95% CI: 18.0–31.0) in the nivolumab-chemotherapy vs. 2.2% (95% CI: 0.6–5.6) in chemotherapy alone]. Adding nivolumab significantly increased the likelihood of achieving a PCR, indicating no residual viable TCs at the time of surgery. This finding suggests that neoadjuvant immunotherapy can effectively reduce tumor burden and potentially facilitate more successful surgical outcomes. Overall, patients receiving nivolumab and chemotherapy had a higher objective response rate of 53.6% (95% CI: 46.0–61.1) compared to chemotherapy alone 37.4% (95% CI: 30.3–45.0%). 4.5% of patients in the nivolumab plus chemotherapy arm had progressive disease vs. 6.1% in the chemotherapy alone arm. In addition, nivolumab plus chemotherapy had a significantly longer median EFS of 31.6 months (95% CI: 30.2–not reached) compared to 20.8 months (95% CI: 14.0–26.7) [5]. Subgroup analysis showed significant benefit in patients (age < 65, HR = 0.57, 95% CI: 0.35–0.93) but not in patients (age ≥ 65, HR = 0.70, 95% CI: 0.45–1.08) and in patients (PD-L1 ≥ 50%, HR = 0.24, 95% CI: 0.10–0.61) but not in patients (PD-L1 1–49%, HR = 0.58, 95% CI: 0.30–1.12; and PD-L1 < 1%, HR = 0.85, 95% CI: 0.54–1.32) (Table 1) [5].

Furthermore, a combination of ICI and chemotherapy was generally safe in CheckMate 816, with manageable toxicity rates similar to previous studies involving nivolumab. Grade 3 or 4 treatment-related adverse events occurred in 33.5% of patients in the nivolumab-chemotherapy group compared to 36.9% in the chemotherapy alone group with the most common grade 3 or 4 treatment adverse event being neutropenia (8.5% with nivolumab plus chemotherapy and 11.9% with chemotherapy alone). The incidence of immune-mediated adverse events was low and only 1.1% of patients had grade 1 or 2 pneumonitis [5]. With regards to surgery, in stage IIIA cases, 16.8% of patients in the nivolumab plus chemotherapy arm did not proceed with definitive surgery, including 8.0% due to disease progression and 1.8% due to adverse events while 24.3% in the chemotherapy-only arm did not proceed with definitive surgery with 13.9% not proceeding due to disease progression and 0.9% due to adverse event. In stage IB–II cases, there was a similar percentage of patients in the nivolumab plus chemotherapy arm (12.3%) vs. the chemotherapy arm (12.9%) who did not proceed to surgery [5]. Adverse events led to 1.1% of surgery cancellations in the nivolumab arm and 0.6% in the chemotherapy arm. Delayed surgery occurred in 20.8% of patients receiving nivolumab and 17.8% receiving chemotherapy though most cases leading to delayed surgery were due to administrative reasons [5].

Finally, CheckMate 816 evaluated the level of ctDNA in a subset of 89 patients. The percentage of patients with ctDNA clearance was higher in the nivolumab with chemotherapy arm (56%, 95% CI: 40–71) vs. chemotherapy alone (35%, 95% CI: 21–51). In addition, there was a significant increase in EFS in patients with ctDNA clearance in both the nivolumab plus chemotherapy group and the chemotherapy-alone group. The percentage of patients with a pathological complete response was higher in those with ctDNA clearance than those without [5]. The promising results from CheckMate 816 provide a strong rationale for the integration of neoadjuvant immunotherapy into the standard treatment protocol for resectable NSCLC [49, 50]. Based on these findings, the NCCN guidelines have been updated to strongly recommend the combination of nivolumab and chemotherapy in patients with tumors measuring 4 cm or larger, or those with node-positive disease, provided no contraindications to ICIs [24].

## Perioperative immunotherapy

Perioperative immunotherapy extends the concept of neoadjuvant treatment by continuing immune modulation into the post-surgical (adjuvant) setting. The goal is to eradicate residual microscopic disease after surgery, which is a critical determinant of long-term outcomes in NSCLC. The rationale is that by stimulating the immune system both before and after surgery, one can maximize the potential for a durable response, reduce recurrence rates, and ultimately improve survival [51].

A key initial perioperative study was the phase II NADIM study looking at stage IIIA resected patients in which patients received neoadjuvant nivolumab and platinum-based chemotherapy and those with R0 surgical resections proceeded to receive 6 months of adjuvant nivolumab. There was a 77.1% 24-month progression-free survival (PFS, 95% CI: 59.9–87.7) and an 81.9% 36-month OS (95% CI: 66.8–90.6) in this study. Moreover, further biomarker analysis showed that low levels of ctDNA prior to treatment were associated with significantly improved PFS and OS while neither PD-L1 staining nor tumor mutation burden were predictive of survival [49, 50]. Furthermore, surgery was cancelled in a greater proportion of patients in the chemotherapy-only arm (31.0%) with not being suitable for surgery (17.2%, 3 of the patients were due to disease extent and 1 was due to recurrent infection) and disease progression (13.7%) being the leading reasons compared to 7.0% in the nivolumab plus chemotherapy arm with no patients having the surgery cancelled due to disease progression and 3 patients (5.2%) not being suitable for surgery due to poor lung function [50].

KEYNOTE-671 is a pivotal study exploring the role of perioperative immunotherapy with pembrolizumab, a PD-1 inhibitor, in patients with resectable NSCLC [43]. This trial encompasses a comprehensive perioperative approach, including pembrolizumab combined with chemotherapy prior to surgery followed by pembrolizumab post-surgery. The study aims to evaluate the impact of this perioperative regimen on DFS and OS, among other outcomes. Initial findings from KEYNOTE-671 suggest that perioperative pembrolizumab, when added to standard chemotherapy, may significantly enhance treatment efficacy in NSCLC, as the EFS at 24 months in the pembrolizumab group was 62.5% compared to 40.6% in the placebo group (HR = 0.58, 95% CI: 0.46–0.72) with a major pathological response occurring in 30.2% (95% CI: 25.7–35.0) in the pembrolizumab group and 11.0% (95% CI: 8.1–14.5) in the placebo group [43]. Subgroup analysis showed significant survival benefits in patients aged < 65 years (HR = 0.53, 95% CI: 0.39–0.71) and patients aged ≥ 65 years (HR = 0.64, 95% CI: 0.46–0.88) and in patients with PD-L1 TPS 1–49% (HR = 0.51, 95% CI: 0.34–0.75) and PD-L1 TPS ≥ 50% (HR = 0.42, 95% CI: 0.28–0.65). In terms of toxicity, no new safety signals were identified with 44.9% of patients in the pembrolizumab group and 37.3% in the placebo group having grade 3 or higher treatment-related adverse events. 5.8% of patients in the pembrolizumab group had grade 3–5 potentially immune-mediated adverse events with 2.0% having pneumonitis and 1.5% having severe skin reactions. 17.9% of patients in the pembrolizumab arm did not go through with surgery, including 6.3% due to adverse events, 3.8% due to progressive disease, and 4.0% due to physician decision compared to 20.5% in the placebo arm including 4.3% due to adverse event, 6.5% due to progressive disease, and 5.0% due to physician decision [43]. Follow-up findings presented at the 2023 European Society for Medical Oncology (ESMO) Congress showed significant OS survival in the pembrolizumab arm (HR = 0.72, 95% CI: 0.56–0.93, *P* = 0.00517) [52]. Median OS was not reached in the pembrolizumab arm vs. 52.4 months in the placebo arm (95% CI: 45.7–not reached) with 36-month OS rates of 71.3% in the pembrolizumab arm vs. 64.0% in the placebo arm [52]. These findings ultimately led to the FDA approval of pembrolizumab in the perioperative setting in October 2023 [53].

**Figure 1:**
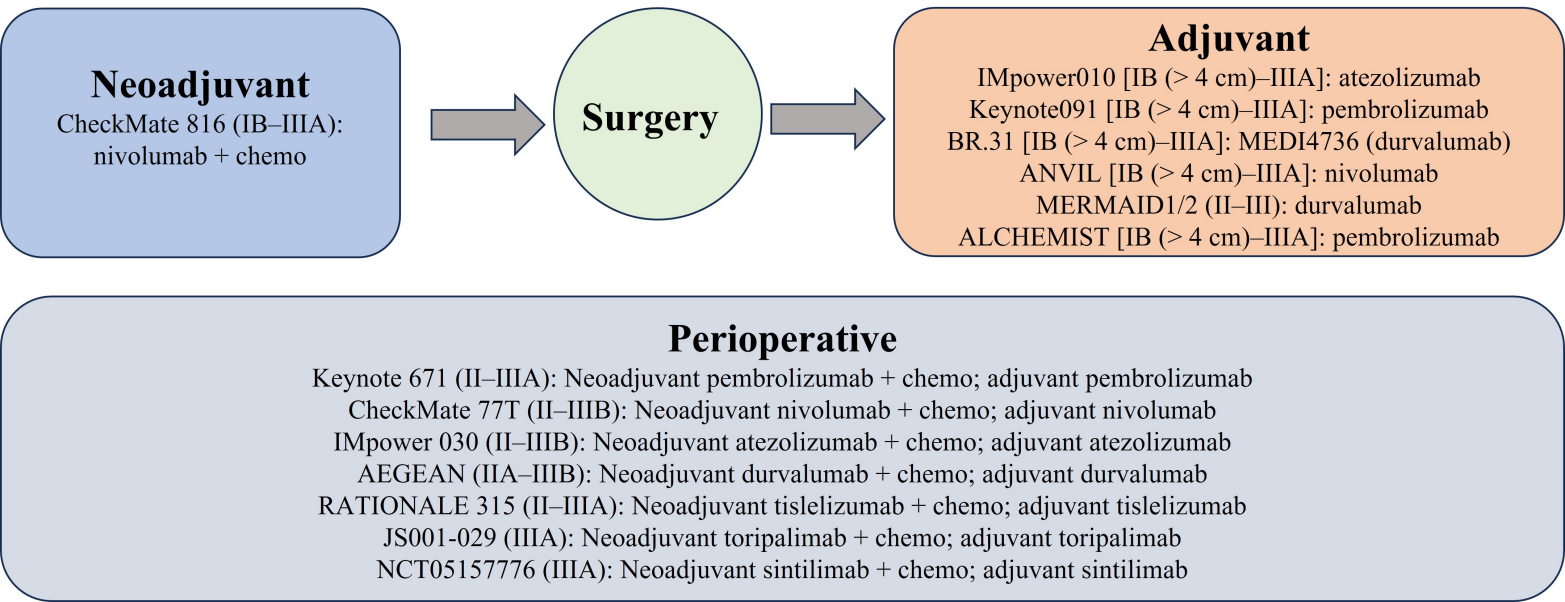
Ongoing phase III clinical trials of perioperative PD-1/PD-L1 inhibitors in operable NSCLC. Chemo: chemotherapy; PD-1: programmed death 1; PD-L1: programmed death-ligand 1; NSCLC: non-small cell lung cancer

Other PD-1/PD-L1 inhibitors have been examined in the perioperative setting. The accompanying clinical trials followed a similar approach, combining immunotherapy with chemotherapy for neoadjuvant treatment, and using immunotherapy alone as an adjuvant therapy (Figure 1, Table 1). Most of these studies evaluated between two and four cycles of neoadjuvant treatment as well as one year of adjuvant immunotherapy. The Checkmate-77T study in which patients with stage IIA–IIIB resectable *EGFR/ALK* wildtype received neoadjuvant nivolumab or placebo plus platinum-doublet chemotherapy and then adjuvant nivolumab or placebo improved EFS in the nivolumab and chemotherapy arm [not reached (95% CI: 28.9–not reached) vs. 18.4 months (95% CI:13.6–28.1), HR = 0.58 (95% CI: 0.42–0.81), *P* = 0.00025]. There was an 18-month EFS of 70.2% in the nivolumab group and 50.0% in the chemotherapy group (HR = 0.58, 97.36% CI: 0.42–0.81). Major pathologic response occurred in 35.4% in the nivolumab group vs. 12.1% in the chemotherapy group. Subgroup analysis showed benefit in all ages [age < 65 years HR = 0.55: (95% CI 0.36–0.85) and age ≥ 65 years HR = 0.61 (95% CI: 0.41–0.91)] and in PD-L1 TPS ≥ 50% (HR = 0.26, 95% CI: 0.12–0.55) but not in PD-L1 TPS < 1% (HR = 0.73, 95% CI: 0.47–1.15) nor PD-L1 TPS 1–49% (HR = 0.76, 95% CI: 0.46–1.25). Similar percentages of patients cancelled surgery (20.1% in the nivolumab arm and 21.6% in the chemotherapy arm) but more cancelled surgery in the chemotherapy arm due to disease progression (9.5% vs. 5.7% in the nivolumab arm). Delays in surgery were similar in both arms (15.7% in nivolumab and 14.2% in chemotherapy) with adverse events as the reason having similar incidence in both arms (3.5% in nivolumab and 3.0% in chemotherapy). Grade 3 or 4 treatment-related adverse events occurred in 32.5% of patients in the nivolumab arm and 25.2% of patients in the chemotherapy arm. Immune-mediated adverse events were uncommon with 5.3% of patients in the nivolumab arm having pneumonitis of any grade and 2.2% with grade 3–5 pneumonitis [7]. The AEGEAN study enrolled stage II–IIIB resected patients to receive neoadjuvant platinum-based chemotherapy plus durvalumab or placebo every 3 weeks for 4 cycles followed by durvalumab or placebo for 12 cycles every 4 weeks post-surgery. Similar to KEYNOTE-671, there was greater efficacy in early data in the durvalumab plus chemotherapy group with 12-month EFS was observed in 73.4% of patients (95% CI: 67.9–78.1) compared to 64.5% of patients in the placebo group (95% CI: 58.8–69.6) [45]. The perioperative phase III Neotorch study in which stage II/III resectable NSCLC patients without *EGFR*/*ALK* alterations in non-squamous NSCLC received neoadjuvant toripalimab or placebo with chemotherapy for 3 cycles followed by toripalimab or placebo monotherapy for 13 cycles every 3 weeks after also showed favorable early findings with significant improvement of EFS in the toripalimab arm (HR = 0.40, 95% CI: 0.277–0.565, *P* < 0.0001) along with a significantly greater major pathologic complete response and pathologic response [54]. Finally, the RATIONALE-315 study compared the efficacy and safety of neoadjuvant tislelizumab or placebo, an anti-PD-1 antibody with chemotherapy, and adjuvant tislelizumab or placebo. This study showed a statistically significant EFS in the tislelizumab arm (HR = 0.56, 95% CI: 0.40–0.79, *P* = 0.0003) and a trend towards OS benefit (HR = 0.62, 95% CI: 0.39–0.98, *P* = 0.193) [46].

Combination therapies are also being considered to augment the early promising results from neoadjuvant ICIs with chemotherapy. The phase II platform NEOSTAR trial in which neoadjuvant ipilimumab and nivolumab and chemotherapy were evaluated showed a major pathologic response rate of 62% (10/16) in patients without *EGFR/ALK* alterations [55]. Other notable combination trials in progress are the SKYSCRAPER-05 trial in which patients receive 4 cycles of neoadjuvant atezolizumab plus tiragolumab, an anti-TIGIT (T cell immunoglobulin and immunoreceptor tyrosine-based inhibitory motif domain) antibody with chemotherapy followed by adjuvant atezolizumab plus tiragolumab for 16 cycles after surgery and the NeoCOAST-2 (NCT05061550) study in which patients will receive either olectumab, a selective anti-CD73 antibody, monalizumab, an anti-NKG2A monoclonal antibody, or AZD-171, an antibody targeting leukemia inhibiting factor (LIF) with platinum-based chemotherapy; or datopotamab deruxtecan, a Trop-2 directed antibody-drug conjugate with durvalumab, or volrustomig, a novel PD-1/CTLA-4 bispecific antibody both as a neoadjuvant treatment and as an adjuvant treatment [56].

Thus far, preliminary data of these studies indicate a favorable safety profile and potential for improved long-term outcomes in NSCLC patients. It fortifies the idea that particularly neoadjuvant chemotherapy and immunotherapy are effective but maturation of this data will be important to help see which patients will gain more from both neoadjuvant chemotherapy and immunotherapy and adjuvant immunotherapy vs. only neoadjuvant chemotherapy and immunotherapy.

## Adjuvant targeted therapy

Over the past 10–15 years, the discovery of gene mutations in NSCLC such as *EGFR*, *ALK*, and other mutations with subsequent development of targeted therapies has dramatically changed the treatment landscape [57–60]. Specifically, in *EGFR* mutations, erlotinib was the first FDA-approved tyrosine kinase inhibitor (TKI) for NSCLC in 2013 for first-line treatment in metastatic *EGFR*-mutated NSCLC based on erlotinib having significant PFS benefit compared to chemotherapy [61]. Subsequently, osimertinib, a third-generation *EGFR* TKI that inhibits both *EGFR* TKI sensitizing and T790M resistant mutations, was studied in metastatic *EGFR*-mutated NSCLC and showed significant OS benefit compared to erlotinib or gefitinib with a median OS of 38.6 months (95% CI: 34.5–41.8) in the osimertinib arm [62]. Similarly, the *ALK* TKIs have also brought significant survival benefits in *ALK*-rearranged, as evidenced first in the PROFILE 1014 study comparing crizotinib to chemotherapy and then in subsequent studies with newer generation TKIs that have improved central nervous system penetration such as alectinib, brigatinib, and lorlatinib [59, 63, 64].

In the adjuvant setting, targeted therapy was first explored with the RADIANT study in *EGFR*-positive stage IB–IIIA NSCLC in which an *EGFR* TKI, erlotinib, was evaluated against a placebo [65]. The study showed mixed results in erlotinib response. For one, the RADIANT study indicated a statistically insignificant difference in DFS between the erlotinib and the placebo group but had a longer DFS compared to the placebo group in the subgroup of *EGFR*-positive patients (HR = 0.61, 95% CI: 0.38–0.98, *P* = 0.039) with no OS benefit (OS HR = 1.09, 95% CI: 0.545–2.161, *P* = 0815) [65]. This suggested that adjuvant-targeted therapy should be further evaluated (Table 2).

Consequently, SELECT, a single-arm phase II study, evaluated adjuvant erlotinib for Stage IA–IIIA *EGFR*-positive NSCLC patients with 2-year DFS of 88% compared to the historical control of 76% from previous cohort data at Memorial Sloan Kettering (*P* = 0.047) [66, 67]. Of note, 65% of the study’s patients were treated with erlotinib after they developed a recurrence of their disease. The medium duration of receiving TKI therapy after retreatment was 13 months, which was similar to the PFS of 13 months for erlotinib when used in newly diagnosed metastatic disease [66, 68].

The ADJUVANT/CTONG 1104 was a randomized controlled phase III study that compared adjuvant gefitinib with adjuvant chemotherapy in stage II–IIIA NSCLC patients who had the *EGFR* exon 19 deletion or exon 21 L858R mutation [69]. In the initial analysis, targeted therapy had a greater median DFS (28.7 months vs. 18.0 months, *P* = 0.0054) [69]. However, in the final analysis, no significant difference in OS was seen after five years 53.2% in the adjuvant erlotinib arm vs. 51.2% in the adjuvant chemotherapy arm (*P* = 0.784) [70]. Subsequently, the EVAN study was a phase II study comparing adjuvant erlotinib and adjuvant chemotherapy in stage IIIA *EGFR*-mutated NSCLC patients [71]. The erlotinib group showed an improved DFS rate compared to chemotherapy at two years (81.35% to 44.62%, *P* = 0.0054). Furthermore, the targeted therapy group’s median survival was 84.2 months vs. 61.1 months for the chemotherapy group (OS HR = 0.318, 95% CI: 0.151–0.670) [72]. This was notable, as this was the first study to demonstrate meaningful OS benefit in adjuvant erlotinib compared to chemotherapy in R0 resected stage III *EGFR*-mutated NSCLC [72].

With the development of osimertinib as the new standard of care for metastatic *EGFR*-mutated NSCLC, the ADAURA study compared osimertinib to placebo in resected stage IB–IIIA *EGFR*-positive NSCLC [9]. The ADAURA study demonstrated a 24-month DFS in osimertinib of 89% to placebo 52% (DFS HR = 0.20, 99.12% CI: 0.14–0.30, *P* < 0.001). Given its increased CNS activity compared to the prior generation of TKIs, the osimertinib arm had fewer CNS recurrences [9]. The final analysis at the five-year interval of the ADAURA study showed an OS rate of 88% in the osimertinib group and 78% in the placebo group (OS HR = 0.49, 95.03% CI: 0.34–0.70, *P* < 0.001) [8]. ADAURA was a practice-changing study that demonstrated the survival benefit of adjuvant osimertinib thus leading to its FDA approval in early-stage *EGFR*-positive NSCLC [8–10].

Despite an increased understanding of adjuvant targeted therapy for NSCLC, there are still some questions that need to be further explored. One question is the optimal timing of adjuvant targeted therapy since the ADJUVANT and EVAN studies received TKIs immediately after resection while the ADAURA study permitted the use of TKIs after adjuvant chemotherapy. Another question is the duration of adjuvant targeted therapy. In the ADAURA study, the osimertinib arm was treated for 3 years compared to the 2 years of TKI treatment in the ADJUVANT and EVAN studies. Thus, future research will need to identify the optimal timing of initiation of targeted therapy post-surgery and the overall duration of adjuvant targeted therapy.

Previous data demonstrated that *ALK* TKIs are the first-line therapy for patients with metastatic *ALK*-positive NSCLC [73, 74]. The benefit of adjuvant targeted therapy has also been investigated in NSCLC patients who have the *ALK* alterations namely with the ALINA study [11, 73–75]. The ALINA study is a randomized controlled phase III study evaluating the use of adjuvant alectinib compared to platinum-based chemotherapy in stage IB–IIIA *ALK*-positive NSCLC who have received complete resection. The initial interim analysis showed a 2-year DFS of 93.8% for the alectinib group and 63.0% for the chemotherapy group (HR = 0.24, 95% CI: 0.13–0.45, *P* < 0.001) and 88.7% and 54.0% at three years respectively [11]. In addition, the HR for CNS disease recurrence or death was 0.22 (95% CI: 0.08–0.58) favoring alectinib [11]. Alectinib in general was relatively well tolerated with no grade 5 adverse events; serious adverse events were reported in 17 patients (13.3%) in the alectinib and all serious events associated with alectinib were resolved [11]. The most commonly reported adverse events were increased creatinine kinase levels (43.0%) and constipation (42.2%). Drug discontinuation occurred in 5.5% of patients in the alectinib compared to 12.5% in the chemotherapy group [11]. The initial analysis of the ALINA study has shown that adjuvant alectinib improved DFS compared to chemotherapy in patients with resected *ALK*-positive NSCLC [11]. The impressive results of the ALINA study led to FDA approval of alectinib in the adjuvant setting in stage IB (≥ 4 cm)–IIIA *ALK*-rearranged NSCLC [76].

**Table 2:** Summary of past and ongoing adjuvant targeted therapy clinical trials

**Trial**	**Phase**	**Stage**	**Mutation**	**Treatment**	**Control**	**Primary endpoint**	**Result**
RADIANT [65]	III	IB–IIIA	EGFR	Erlotinib × 2 years	Placebo	DFS	50.5 months erlotinib vs. 48.5 months placebo; HR = 0.90, 95% CI: 0.74 to 1.10
SELECT [66]	II	IB–IIIA	EGFR	Erlotinib × 2 years	None	DFS	88% (96% stage I, 78% stage II, 91% stage III) vs. historic data of 76%
ADJUVANT/CTONG1104 [69]	III	II–IIIA	EGFR	Gefitinib × 2 years	Chemotherapy	DFS	28.7 months gefitinib (95% CI: 24.9–32.5) vs. 18.0 months chemotherapy (95% CI: 13.6–22.3); HR = 0.60, 95% CI: 0.42–0.87, *P* = 0.0054
EVAN [71]	II	IIIA	EGFR	Erlotinib × 2 years	Chemotherapy	DFS	81.4% (95% CI 69.6–93.1) erlotinib vs. 44.6% (26.9–62.4) chemotherapy
ADAURA [9]	III	IB–IIIA	EGFR	Osimertinib × 3 years	Placebo	DFS	90% (95% CI: 84–93) osimertinib vs. 44% (95% CI: 37–51); HR = 0.17, 99.06% CI: 0.11–0.26, *P* < 0.001
EVIDENCE [77]	III	II–IIIA	EGFR	Icotinib × 2 years	Chemotherapy	DFS	47.0 months icotinib (95% CI: 36.4–NYR) vs. 22.1 months chemotherapy (95% CI: 16.8-30.4); HR = 0.36, 95% CI: 0.24–0.55, *P* < 0.0001
ALCHEMIST-EGFR [75]	III	IB–IIIA	EGFR	Erlotinib × 2 years	Placebo	OS	N/A
ICOMPARE [78]	II	II–IIIA	EGFR	Icotinib × 1 year	Icotinib × 2 years	DFS	32.9 months icotinib for 1 year (95% CI: 26.6–44.8) vs. 48.9 months for 2 years (95% CI: 33.1–70.1); HR = 0.51; 95% CI: 0.28–0.94, *P* = 0.0290
FORWARD (NCT04853342)	III	II–IIIA	EGFR	Furmonertinib	Placebo ± chemotherapy	DFS	N/A
NCT04687241	III	II–IIIB	EGFR	Almonertinib	Placebo	DFS	N/A
NCT05241028	II	IB–IIIA	EGFR	Ensartinib × 3 years	None	DFS	N/A
ALINA (NCT03456076) [11]	III	IB–IIIA	ALK	Alectinib × 2 years	Chemotherapy	DFS	93.8% alectinib vs. 63.0% chemotherapy (HR = 0.24, 95% CI: 0.13–0.43, *P* < 0.001)
ALCHEMIST-ALK [75]	III	IB–IIIA	ALK	Crizotinib × 2 years	Placebo	OS	N/A

DFS: disease-free survival; OS: overall survival; mPR: major pathological response; ORR: objective response rate; NYR: not yet reached; EGFR: epidermal growth factor receptor; ALK: anaplastic lymphoma kinase; HR: hazard ratio; CI: confidence interval; N/A: not applicable

## Neoadjuvant targeted therapy

Since the literature demonstrated a survival benefit for patients with *EGFR/ALK*-positive NSCLC after they received adjuvant targeted therapy, this inspired researchers to investigate the utility of neoadjuvant targeted therapy (Table 3).

ESTERN, a single-center phase II study, evaluated neoadjuvant erlotinib in patients with stage IIIA N2 NSCLC who had a positive *EGFR* mutation on exon 19 or 21. After receiving neoadjuvant erlotinib, 93.8% of the 16 patients successfully had an R0 resection [79]. Upon final analysis, the radical resection rate for the group was 68.4% (13/19), and the objective response rate was 42.1% [80]. Moreover, this study showed that neoadjuvant *EGFR* TKI was well tolerated from a side effect profile and may improve outcomes after surgical resection [80].

The EMERGING-CTONG 1103 study, a randomized phase II trial, compared neoadjuvant erlotinib 150 mg daily up to 42 days and adjuvant erlotinib up to 12 months with gemcitabine plus cisplatin (2 cycles of neoadjuvant therapy and up to 2 cycles of adjuvant therapy) chemotherapy in patients with stage IIIA N2 *EGFR*-positive NSCLC [81]. The primary endpoint was ORR, and the study did not quite meet its endpoint but there was a trend towards improved ORR in neoadjuvant erlotinib vs. gemcitabine and cisplatin chemotherapy (54.1% vs. 34.3%, ORR: 2.26, 95% CI: 0.87–5.84; *P* = 0.092). There also was a significant improvement in median PFS (21.4 months vs. 11.4 months, HR = 0.39, 95% CI: 0.23–0.67, *P* < 0.001) [81]. With the updated analysis, neoadjuvant erlotinib continued to demonstrate an improved PFS (21.5 months vs. 11.4 months, HR = 0.36, 95% CI: 0.21–0.61, *P* < 0.001) but not a significant difference in OS (42.2 months in the erlotinib arm vs. 36.9 months in the chemotherapy arm, HR = 0.83, 95% CI: 0.47–1.47, *P* = 0.513) [82].

Researchers have been designing newer clinical trials, including neoadjuvant *EGFR* TKIs with chemotherapy for patients with *EGFR*-positive NSCLC. A single-arm phase IIB trial of stage IIA–IIIB *EGFR* exon 19 and/or 21 mutations with 88 patients had an ORR of 71.1% (95% CI: 55.2–83.0) in 38 patients who completed 6 weeks of osimertinib along with 93.8% undergoing successful R0 resection [83]. Examples of these trials include both the NeoADAURA and the NOCE01 (NCT05011487) [84]. These studies may open a new avenue in the management of NSCLC by expanding the use of neoadjuvant *EGFR*-targeted therapy.

Similarly, there is no FDA approval for neoadjuvant TKIs in patients with resectable *ALK*-positive NSCLC due to the lack of trial data. Several trials (SAKULA, RTOG 1306, ARM) were designed to evaluate the efficacy of neoadjuvant *ALK* TKIs, but they were terminated early due to the slow accrual of patients [85, 86]. For the SAKULA trial, there were 7 patients who received neoadjuvant ceritinib and had a 100% reported response rate. After resection, the major pathological response was 57% [86]. RTOG 1306 compared crizotinib followed by chemoradiation to chemoradiation alone in 16 patients. The crizotinib group’s complete or partial response was 67%, and the chemoradiation group’s was 76% [85]. The lack of improvement in the RTOG 1306 trial compared to the SAKULA trial could be explained that RTOG 1306 used crizotinib, a 1st generation *ALK* TKI, instead of ceretinib, a 2nd generation *ALK* TKI, in the SAKULA study [85, 86].

Additionally, there was a small study where neoadjuvant crizotinib was evaluated in patients with TXN2M0 disease. Out of 11 patients in the study, 10 achieved a partial response after neoadjuvant crizotinib [87]. It was also seen that 10 (91%) of the patients received an R0 resection, and 2 achieved a complete pathological response. 6 patients developed recurrence of their disease, and 5 of them were restarted on crizotinib [87]. Regardless of this study’s small sample size, it showed that a neoadjuvant *ALK* TKI may provide a benefit to *ALK*-positive NSCLC patients.

Ongoing trials are evaluating the efficacy of neoadjuvant *ALK*-targeted therapy. One such trial is ALNEO (NCT05015010), which is a single-arm study that evaluates neoadjuvant alectinib in patients with resectable stage III *ALK*-positive NSCLC [88]. Another is NAUTIKA-1, a single-arm study, where *ALK*-positive patients with resectable stage IB–IIIA receive neoadjuvant alectinib undergo surgery followed by up to 4 weeks of platinum-based chemotherapy then continue adjuvant alectinib (NCT04302025). These ongoing clinical trials provide an exciting avenue to expand our understanding of neoadjuvant *ALK*-targeted therapy but they also face a similar challenge as the prior studies: accrual of patients given the rare incidence of *ALK* mutations. Although the data is limited for neoadjuvant targeted therapy thus far, there have been promising results indicating a potential benefit of neoadjuvant targeted therapy for patients with NSCLC who express a driver mutation.

**Table 3:** Summary of past and ongoing neoadjuvant targeted therapy clinical trials

**Trial**	**Phase**	**Stage**	**Mutation**	**Treatment**	**Control**	**Primary endpoint**	**Result**
ESTERN [79]	II	IIIA	EGFR	Erlotinib × 2 years	None	Radical resection rate	60%
NCT00600587 [89]	II	IIIA (N2)	EGFR	Erlotinib × 6 weeks	Chemotherapy	Response rate	58.3% erlotinib vs. 25.0% chemotherapy (*P* = 0.18)
NCT01217619 [80]	II	IIIA	EGFR	Erlotinib × 8 weeks	None	Radical resection rate	68.4%
EMERGING-CTONG 1103 [81]	II	IIIA (N2)	EGFR	Erlotinib × 6 weeks	Chemotherapy	ORR	54.1% erlotinib vs. 34.3% chemotherapy (95% CI: 0.87–5.84, *P* = 0.092)
NCT03203590	III	II–IIIA	EGFR	Gefitinib × 8 weeks	Chemotherapy	DFS	N/A
NeoADAURA [84]	II	II–IIIA	EGFR	Osimertinib ± chemotherapy	Chemotherapy	mPR	N/A
NCT03433469 [90]	II	I–IIIA	EGFR	Osimertinib	None	mPR	15%
ChiCTR1800016948 [83]	II	IIA–IIIB	EGFR	Osimertinib × 6 weeks	None	ORR	71.1% (95% CI: 55.2–83.0)
NOCE01 (NCT05011487)	II	IIIA (N2)	EGFR	Osimertinib × 60 days + chemotherapy × 2 cycles	None	Lymph node clearance rate	N/A
NCT03349203	II	IIIB, oligometastatic	EGFR	Icotinib × 8 weeks as neoadjuvant therapy, then 2 years as adjuvant therapy	None	ORR	N/A
NCT03749213	II	IIIA–N2	EGFR	Icotinib × 8 weeks as neoadjuvant therapy, then for 2 years as adjuvant therapy	None	ORR	N/A
NCT04965831	II	IIIA–IIIB (N1–N2)	EGFR	Furmonertinib × 8 weeks as neoadjuvant therapy, then 2 years as adjuvant therapy	None	ORR	N/A
NCT05241028	II	IB–IIIA	EGFR	Ensartinib × 3 years	None	DFS	N/A
SAKULA [86]	II	II–III	ALK	Ceritinib × 12 weeks	None	mpR	57% (95% CI: 18–90)
RTOG 1306 (NCT01822496)	II	III	ALK	Crizotinib × 12 weeks	Placebo	PFS	N/A
ARM (NCT03088930)	II	IA–IIIA	ALK, ROS1, MET	Crizotinib × 6 weeks	None	ORR	N/A
ALNEO [88]	II	III	ALK	Alectinib × 8 weeks, adjuvant alectinib × 96 weeks	None	mPR	N/A
NAUTIKA-1 (NCT04302025)	II	IB–III	ALK, ROS1, NTRK, BRAF V600E, RET	Alectinib × 8 weeks, followed by adjuvant alectinib × 104 weeks	None	mPR	N/A

DFS: disease-free survival; OS: overall survival; mPR: major pathological response; ORR: objective response rate; EGFR: epidermal growth factor receptor; ALK: anaplastic lymphoma kinase; N/A: not applicable; PFS: progression free survival; CI: confidence interval

## Discussion

Over the past few years, advances in immunotherapy and targeted therapy have made significant strides in translating to clinical benefits of these therapies in the early-stage resectable setting. However, while these findings are quite exciting in improving the long-term outcomes of early-stage NSCLC patients, there are numerous issues that remain unanswered.

The first challenge is understanding the clinical implications of major pathologic and pathologic complete responses in patients. Studies predating the recent perioperative immunotherapy studies have suggested that this endpoint may be a surrogate for improved prognosis particularly in neoadjuvant chemotherapy-only studies [48]. In an early analysis of Checkmate 816, it appears that there is an association between neoadjuvant immunotherapy and major pathologic response in terms of EFS, as 2-year EFS rates were 90%, 60%, 57%, and 39% for patients with 0–5%, > 5–30%, > 30–80%, and > 80% residual volume of tumor post-surgery, respectively [91]. Another meta-analysis consisting of KEYNOTE-671, NADIM II, and AEGEAN showed that that major pathological complete response was a significant variable in OS, but another recent analysis of neoadjuvant trials evaluating whether pathologic complete response and major pathologic response showed a robust 2 years EFS correlation but no OS correlation [92, 93]. Thus, moving forward, it remains to be seen if the pathologic complete response and major pathologic response will be reliable surrogates for OS given the lack of maturity of current OS data and the possibility of study crossover in these studies.

The ability to choose between both neoadjuvant and adjuvant therapy options also presents a new challenge to interpreting major and complete pathologic responses in the context of receiving additional systemic therapy after surgery in patients who do achieve a major pathologic response. One test that could become valuable in the setting of perioperative NSCLC is ctDNA testing. In the NADIM-2 trial, low pretreatment levels of ctDNA were associated with significantly improved PFS and OS while in CheckMate 816 exploratory analysis showed that patients achieving clearance of their ctDNA by the beginning of the third cycle had higher pathological complete response rate than patients whose ctDNA did not clear [5]. A prospective study evaluating early-stage NSCLC patients showed the utility of using serial ctDNA in the presurgical and postsurgical setting in a longitudinal manner, as detectable ctDNA in any of these settings was associated with inferior recurrence-free survival [94]. It is also important to note that in the IMPower010 adjuvant atezolizumab study regardless of ctDNA status, adjuvant atezolizumab was linked to improved DFS in PD-L1+ subgroups so ctDNA may not be the only marker in determining prognosis in perioperative studies [95]. Nevertheless, ctDNA testing could be very valuable in the perioperative setting, but continued studies evaluating ctDNA as survival data matures and streamlined testing for a reliable assay will be important moving forward along with increased sensitivity of such ctDNA testing.

Another question moving forward is understanding the benefits of neoadjuvant vs. adjuvant therapies. Currently, many of the perioperative immunotherapy studies are showing early success in DFS of upwards to 2 years in the neoadjuvant and adjuvant setting and increased pathologic complete response rates. However, it will be interesting to see how these results compare once the data matures and we see whether studies such as KEYNOTE 671 with a neoadjuvant and adjuvant approach have a notable survival difference compared to a neoadjuvant only approach as seen in CheckMate 816 or an adjuvant only approach as seen in IMPower010. However, while we can consider pathologic complete response and ctDNA clearance as possible prognostic tools and wait for the OS data to mature, we need more information to better predict which patients will respond to perioperative immunotherapy. Interestingly, other biomarkers such as PD-L1 tumor proportion score that is frequently used in adjuvant immunotherapy such as IMPower010 somehow do not seem to correlate thus far in studies such as CheckMate 816 [2, 5]. While some may consider this as PD-L1 not being a good marker for studies involving neoadjuvant immunotherapy, it suggests more that we need more comprehensive and sophisticated biomarkers to help better make decisions involving immunotherapy or targeted therapies. With advances in artificial intelligence (AI) and machine-learning, we should consider creating models based on gene signatures that portend a robust tumor immune microenvironment and use this to help make decisions along with radiomics-based AI to help predict the tumor microenvironment or use of pathology-based AI looking at density of CD3+ and CD8+ T cells [96–98]. Another novel idea is plasma proteomic-based models such as PROphet that can be used to predict both therapeutic benefits and immune-related adverse events [99]. Thus, the hope is that the implementation of technological advances that can incorporate signatures of many genes associated with immunotherapy response will provide a much better-informed decision for the clinician when deciding on immunotherapy use.

Another critical issue is balancing toxicity and efficacy. There are still approximately 15% of patients across trials receiving neoadjuvant therapy in which patients have either disease progression, adverse events, or worsening lung function, unresectability, or patient refusal. With additional studies like NEOSTAR, SKYSCRAPER-05, and NeoCOAST-2 where patients are receiving dual immunotherapy agents or antibody-drug conjugate, the concerns about immune-related adverse events and adverse-related events secondary to an antibody-drug conjugate are heightened [55, 56]. While there may be an increase in major pathologic complete response as evidenced in the NEOSTAR study, there is also a potentially significant increase risk of immune-related adverse events which could lead to patients not being able to proceed with surgical resection [55].

With perioperative immunotherapy trials, there are other factors to consider beyond the depth of response. Other factors that can get overlooked particularly during the analysis of these studies is the consideration of comorbidities such as autoimmune disease, which can be exacerbated with the use of ICI [100]. Also, while single-agent ICI has been well tolerated in general, Eastern Cooperative Oncology Group (ECOG) performance status should still be considered particularly when considering the combination of these agents with chemotherapy or a second checkpoint inhibitor [101]. Furthermore, drug interactions with these regimens should not be discounted, as even common medications used a pre-medications such as proton pump inhibitors or H2-receptor antagonists may impact the efficacy of the drug [102].

While ADAURA and initial results from the ALINA studies have been practice-changing in treating patients in the adjuvant setting, there remain notable questions. One question is the optimal timing of adjuvant targeted therapy and whether to use adjuvant chemotherapy since patients in the ALINA study received alectinib immediately after resection while the ADAURA study permitted the use of TKIs after adjuvant chemotherapy. Next, the duration of treatment is at the forefront, as in the ADAURA trial patients received 3 years of adjuvant osimertinib and in the ALINA trial patients received 2 years of adjuvant alectinib [9, 103]. Of note, the ADJUVANT/CTONG 1104 study had post hoc analysis evaluating receiving gefitinib vs. vinorelbine and cisplatin chemotherapy in patients with resected *EGFR*-mutation positive stage II–IIIA NSCLC and showed that gefitinib compared to vinorelbine and cisplatin did slow down the median time to disease recurrence, but that recurrence did increase at a constant rate 12 months post-surgery, and unfortunately, gefitinib did not have a lower percentage of CNS recurrence compared to vinorelbine and cisplatin [104]. The TARGET (NCT05526755) study is a phase II single-arm study looking at 5 years of adjuvant osimertinib use in stage II–IIIB NSCLC and may provide some clues as to whether a longer duration than 3 years is needed or if tools like ctDNA clearance to detect MRD may help guide the decision on the duration between 3, 4, or 5 years [105]. Another aspect that has been considered but with no results yet in a phase III study is the neoadjuvant use. The EMERGING-CTONG 1103 study was a randomized controlled phase II study looking at neoadjuvant erlotinib and chemotherapy in *EGFR*-mutated stage IIIA (N2) NSCLC and while there was a longer PFS, this did not lead to an OS benefit [106]. The NeoADAURA is a phase III study comparing neoadjuvant osimertinib with or without chemotherapy to chemotherapy alone in patients with resectable, *EGFR*-mutated, stage II–IIIB NSCLC, with a major pathological response as the primary end point [107]. It remains to be seen if perioperative osimertinib may be different, as some of the shortcomings to earlier generation TKIs may be associated with CNS recurrence and osimertinib and alectinib have far superior CNS penetration to previous studies.

Finally, the financial implications cannot be overlooked when considering the idea of providing perioperative treatments to minimize the risk of recurrence of patients’ lung cancer treatment. For example, the course of adjuvant immunotherapy for 1 year of atezolizumab as per the IMPower010 study costs approximately $163,221.12 [$10,201 per 1 time (1,200 mg dose) for 16 cycles] while the cost for 3 cycles of neoadjuvant immunotherapy as in the Checkmate 816 study costs approximately $44,768.16 [$14,922.72 per 1 time (360 mg dose) for 3 cycles] [108]. Meanwhile, another study on adjuvant osimertinib cost-effectiveness showed that it would cost $317,119 per quality-adjusted life years (QALY)-gained for osimertinib to be considered cost-effective [109]. While the value of life is immeasurable, financial strain is a huge mental and physical burden to patients and their families and if there is a marginal benefit, the financial benefit for this marginal benefit needs to be seriously considered.

Moving forward, there is considerable excitement and anticipation regarding improvements in the management of early-stage NSCLC to help decrease the high risk of recurrence with the use of immunotherapy and targeted therapies and tools and benchmarks such as ctDNA and pathologic complete response that may better guide decision making for systemic therapies. The primary challenge, however, will be weighing the benefits of such treatments while not causing overwhelming toxicity particularly as newer combination immunotherapies or new classes of drugs such as antibody-drug conjugates are developed and employed.

## Conclusions

While early-stage NSCLC is treatable and curable, the risk of recurrence is high. With the implementation of immunotherapy and targeted therapy, we have been able to have significant benefits initially in adjuvant use in immunotherapy with the IMPower010 trial using adjuvant atezolizumab and then in the targeted therapy setting with the ADAURA trial using adjuvant osimertinib and with the ALINA trial using adjuvant alectinib. In early returns, we see encouraging results in the neoadjuvant setting with Checkmate 816 with chemotherapy and nivolumab and in the perioperative setting OS benefit in KEYNOTE-671 with perioperative chemotherapy and pembrolizumab. We look forward to better understanding the implications of these findings as OS data matures and we continue to work on finding prognostic biomarkers and tools such as pathologic complete response and ctDNA that will better shape our decision-making in systemic therapy in perioperative NSCLC patients.

## Abbreviations

AI: artificial intelligence
ALK: anaplastic lymphoma kinase
CI: confidence interval
ctDNA: circulating tumor DNA
CTLA-4: cytotoxic T lymphocyte antigen 4
DFS: disease-free survival
EFS: event-free survival
EGFR: epidermal growth factor receptor
FDA: Food and Drug Administration
HR: hazard ratio
ICI: immune checkpoint inhibitor
MRD: minimal residual disease
NCCN: National Comprehensive Cancer Network
NSCLC: non-small cell lung cancer
ORR: overall response rate
OS: overall survival
PCR: pathological complete response
PD-L1: programmed death ligand 1
PFS: progression-free survival
TKI: tyrosine kinase inhibitor
TPS: tumor proportion score
VPI: visceral pleural invasion

## Declarations

### Copyright

© The Author(s) 2024.
